# Supplementary material for: CLOUD: A Scalable and Physics-Informed Foundation Model for Crystal Representation Learning
Source: Nat Commun. 2026 Mar 17;17:4074. doi: 10.1038/s41467-026-70467-3 (PMC13144728; doi:10.1038/s41467-026-70467-3)
Supplement: Supplementary file 1 — Supplementary Information [file 41467_2026_70467_MOESM1_ESM.pdf]

Supplementary Information for CLOUD: A Scalable and Physics-Informed Foundation Model for Crystal Representation Learning

Changwen Xu[1], Shang Zhu[1], Venkatasubramanian Viswanathan[1,2*]

[1]Department of Mechanical Engineering, University of Michigan.
[2]Department of Aerospace Engineering, University of Michigan.

*Corresponding author(s). E-mail(s): venkvis@umich.edu;
Contributing authors: changwex@umich.edu; shangzhu@umich.edu;

S1 Limitations and Future Work

While CLOUD shows competitive performance in crystal representation learning and property prediction, it does encounter limitations and potential improvements for future explorations. One limitation is that our model requires a large amount of pre-training data to achieve SOTA performance in many of the downstream tasks according to the scaling law we fitted, whereas such a dataset has not yet been built. Currently, the largest crystal database we can build is the one collected from OPTIMADE which includes ∼6.3M unique crystal structures. Compared to molecular science in which ∼50 billion unlabeled SMILES of molecules can be easily obtained [1–3], obtaining such a large number of crystal structures is challenging despite the large design space of crystals. Not all combinations of atoms can form valid crystal structures [4] as many potential configurations are inherently unstable or chemically incompatible. Recently, a lot of machine learning models have been proposed for crystal generation [5–8], however, none of them have pushed the data size to the order of billions. Recently, OMat24 dataset [9] was released which contains over 100 million DFT calculations for solid-state materials, which is a huge contribution to the research community to advance materials science development with AI. In future work, we plan to comprehensively integrate existing datasets like OMat24 and diverse generation approaches for crystal structure generation, ranging from rule-based methods to data-driven approaches. We will examine how the expanded, synthetic dataset impacts the model performance in both pre-training scaling and downstream predictive accuracy.

Another limitation lies in the representation itself: the representation we design is built on symmetry, whereas symmetry does not capture everything. For instance, while the space group, Wyckoff positions, and stoichiometry remain unchanged during relaxation, the atomic positions and cell parameters undergo significant variations. As a result, CLOUD is unable to leverage the MPtrj dataset, which comprises over 1.5 million crystal structures from relaxation trajectories, unlike many other models on the leaderboard [10, 11]. In addition, the representation does not work for amorphous materials which lack the long-range order and symmetry that crystalline materials exhibit, thus limiting the versatility of CLOUD in handling a broader range of materials. Furthermore, some Wyckoff positions include free variables, meaning the representation encodes an ensemble of materials. As a result, it may not distinguish between materials with different physico-chemical properties that share the same prototype but have different atomic positions. In addition, two further limitations deserve mention. First, although the SCOPE representation is invariant to symmetry operations by design, it is not invariant to permutations of the atom list in the input structure. Since Wyckoff labels are assigned in the order atoms are provided, permuting the atoms in a CIF can lead to different sequences, which is undesirable given that language models are sensitive to token order, and in future work we plan to solve it by enforcing a canonical ordering of Wyckoff sites (e.g., sorted by site symmetry, starting from 1a) and permuting elements accordingly. Second, while SCOPE distinguishes compounds with different element–Wyckoff assignments, it cannot fully resolve cases where multiple elements partially occupy the same Wyckoff position. In such situations, only the combined occupancy is encoded, rather than the specific site assignments of each element. This limitation becomes important for modeling disorder and site mixing, and extending SCOPE in this direction represents a promising direction for future work. In future work, we plan to systematically investigate the design of string representations for crystals which enables effective encoding of more information besides symmetry and composition, while building the multi-modal framework for crystalline materials as an alternative to incorporate comprehensive structural information.

S2 Implementation Details

S2.1 Additional Dataset Information

MatBench [12] is a benchmark test suite that contains 13 tasks using data from density functional theory-derived and experimental sources. We use 8 out of 13 tasks for benchmarking as they are regression tasks and provide the crystal structures to build the SCOPE representation.

MatBench Discovery [11] is a comprehensive resource designed to evaluate machine learning models for materials discovery, particularly for predicting the thermodynamic stability of materials. The dataset aims to reflect practical challenges in the discovery process by requiring predictions based on unrelaxed crystal structures, which avoid reliance on expensive DFT calculations. The models are trained on customized training data and tested on WBM dataset [13] which contains ∼257K OOD crystal structures generated by systematically substituting elements in pre-existing structures from the

Materials Project (MP). This dataset is heavily composed of ternary phases, with a significant fraction of transition metals and metalloids, offering a broader chemical diversity than the MP dataset. The WBM is designed to challenge models to perform OOD predictions, making it a demanding benchmark for stability.

UnconvBench [14] consists of unconventional crystal structures including 2D crystals, metal-organic frameworks (MOFs), defected crystals. The benchmark contains various crystals with irregular and complex long-range, whereas such crystalline systems are rarely observed in highly ordered traditional crystals. Hence, UnconvBench provides complementary insights to existing benchmark datasets, offering a broader perspective on evaluating model performance on unconventional materials (which are in fact conventional in the real world).

C_v and U datasets [15] consist of heat capacity (C_v) and phonon internal energy (U) data for a diverse set of materials. The dataset is derived from DFT calculations and provides key thermodynamic properties at 300K. The phonon properties heavily depend on the periodicity and bonding strength of the crystal structure, making this dataset a crucial benchmark for evaluating machine learning models' ability to capture long-range interactions and structural periodicity. The dataset has been used in previous studies to analyze the limitations of graph neural networks in predicting phonon-related properties and has been shown to benefit from explicit periodicity encoding.

S2.2 Training Details

The hyperparameters used for training CLOUD are summarized in Table S1. A list of values is shown in the table if the hyperparameter takes different values for different tasks. The model architecture is fixed to 12 hidden layers and 12 attention heads in each layer for the BERT part in CLOUD when used for benchmarking, while we experiment with smaller models in order to fit the scaling law. The number of hidden layers is fixed to 1 for experiments on MatBench for a fair comparison, while we experiment with up to 3 hidden layers in the prediction head on MatBench Discovery and UnconvBench for optimal performance as we do not need to compare with MatInFormer or SLICES-BERT on those two benchmarks. The hyperparameters that are used in experiments for pre-training and fine-tuning are also listed in Table S1.

S2.3 Baseline

We summarize the baseline models used for benchmarking in Table S2. The baselines cover a variety of models:

- Voronoi RF directly uses chemical descriptors as features for input.
- Roost, Finder, and CrabNet use composition only as input.
- coGN, coNGN, ALIGNN, MEGNet, CGCNN, CGCNN+P, and BOWSR build graphs from crystal structures and learn the mapping from the structure to target properties.
- SevenNet, MACE, CHGNet, and M3GNet are GNNs trained to serve as universal machine learning-based interatomic potentials (MLIPs).

Table S1: Hyperparameters of CLOUD.

Hyperparameter	Value
# of hidden layers in BERT	{1,3,6,12}
# of attention heads in BERT	{1,4,12}
# of hidden layers in MLP	{1,2,3}
max position embeddings	64
block size	64
embedding size	768
# of hidden layers in MLP	{1,2,3}
hidden layer width in MLP	768
activation function in MLP	SiLU
pre-train epochs	50
pre-train learning rate	1e-4
pre-train batch size	2048
pre-train weight decay	0.0
pre-train optimizer	AdamW
pre-train scheduler	linear warmup and cosine decay
pre-train warm-up ratio	0.05
fine-tune epochs	{50,100,200}
fine-tune learning rate	{1e-4,5e-5}
fine-tune batch size	{32,64,128,512}
fine-tune weight decay	{0.0, 0.0001, 0.01}
fine-tune optimizer	AdamW
fine-tune scheduler	linear warmup and cosine decay
fine-tune warm-up ratio	{0.05,0.1}
fine-tune # of freezing-encoder epochs	{0,5,10}

- CrysToGraph is a transformer-based geographic model for learning both local and long-range information from graphs.
- MatInFormer, Wrenformer, and SLICES-BERT are built on a transformer architecture and take sequences as input, same as our model CLOUD.

S2.4 Evaluation Metrics

Our evaluation strategies follow the ones adopted by the benchmark leaderboards. We use MAE as the metric for regression tasks in MatBench and UnconvBench. MatBench Discovery leaderboard provides results for multiple metrics: MAE, RMSE, and R^2 for energy above convex hull prediction, and F1, accuracy, precision, true positive rate (TPR), and true negative rate (TNR) for stability classification. In particular, [11] propose discovery acceleration factors (DAF) quantifies how much faster a machine learning model can identify stable materials compared to random selection. The expression of DAF is given as follows:

$$\mathrm{DAF} = \frac{\mathrm{Precision}}{N_{\mathrm{stable,true}}/N_{\mathrm{total}}} \tag{1}$$

where $N_{\mathrm{stable,true}}$ and N_{total} are the number of stable materials and the total number of materials in the dataset, respectively.

Table S2: Summary of baseline models used in this work.

Model	Type	Architecture	Source
coGN	Structure-based	GNN	[16]
coNGN	Structure-based	GNN	[16]
ALIGNN	Structure-based	GNN	[17]
CGCNN	Structure-based	GNN	[18]
CrabNet	Structure-agnostic	Transformer	[19]
Finder	Structure-agnostic	GNN	[20]
Roost	Structure-agnostic	GNN	[21]
Wrenformer	Coordinate-free	Transformer	[11]
MatInFormer	Coordinate-free	Transformer	[22]
SLICES-BERT	Coordinate-free	Transformer	[23]
SevenNet	Structure-based (MLIP)	GNN	[24]
MACE	Structure-based (MLIP)	GNN	[25]
CHGNet	Structure-based (MLIP)	GNN	[10]
M3GNet	Structure-based (MLIP)	GNN	[26]
MEGNet	Structure-based	GNN	[27]
CGCNN+P	Structure-based	GNN	[28]
BOWSR	Structure-based	GNN	[29]
Voronoi RF	Feature-based	Random Forest	[30]
CrysToGraph	Structure-based	Graph Transformer	[14]

We also provide the results for ROC-AUC in order to remove the sensitivity of the classification metrics to the choice of thresholds.

For C_v and U datasets, we use MAE/MAD as the metric, consistent with the original literature [15]. First proposed in [17], the metric normalizes MAE with the mean absolute deviation (MAD) so that model performance could be compared across datasets with different units and scales:

$$\text{MAE/MAD} = \frac{\sum |y_i - y_{i,\text{true}}|}{\sum |y_{i,\text{true}} - \overline{y}|} \tag{2}$$

where y_i and $y_{i,\text{true}}$ are the predicted value and the true value of the ith data point, and $\overline{y}$ is the mean of the true values in the dataset. Usually, a model will be considered as a good predictive model if MAE/MAD < 0.2 [17, 31].

S3 Additional Results

S3.1 MatBench

We provide the MAE for each model on the eight regression tasks from MatBench [12] in Table S3. We provide the average MAE as well as the standard deviation on five folds for each task. CLOUD surpasses all the other structure-agnostic and coordinate-free models on 7 out of 8 tasks on MatBench. Furthermore, CLOUD achieves state-of-the-art results on jdft2d and dielectric datasets.

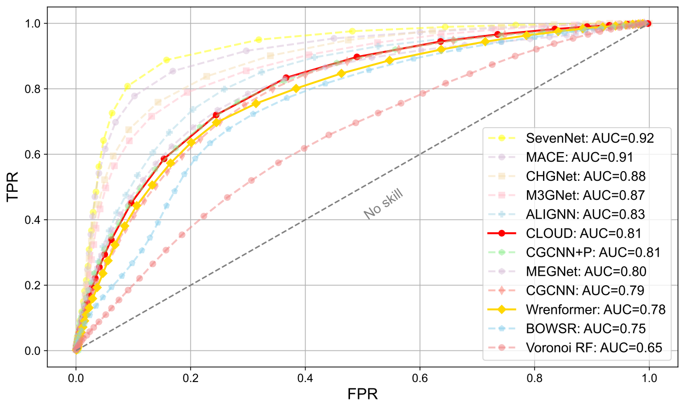

Fig. S1: Receiver operating characteristic (ROC) curves for each model evaluated on MatBench Discovery. False positive rate (FPR) on the x-axis is the fraction of unstable structures classified as stable. True positive rate (TPR) on the y-axis is the fraction of stable structures classified as stable. Area under curve (AUC) scores are calculated and presented in descending order. More models are included in the plot compared to Figure 2**c**.

S3.2 MatBench Discovery

We train the model on Materials Project [32] formation energy data from the v2022.10.28 MP release, then make predictions for the unrelated structures in the WBM dataset [13] which are generated via elemental substitution of MP source structures so that the generated structures are not included in the training set. When evaluating the models on MatBench Discovery, we record the classification results under varied thresholds and plot the receiver operating characteristic (ROC) curve for CLOUD and the major structure-based models that are trained on MatBench. We plot the ROC curves with more models included in Figure S1 compared to Figure 2**c**. Though not comparable to machine-learned interatomic potentials (MLIPs) at this stage, CLOUD still shows solid performance compared with CGCNN, MEGNet, Wrenformer, etc.

We further examine the other metrics listed in Table S4. The regression metrics of CLOUD are again second to ALIGNN while outperforming CGCNN and MEGNet. The classification metrics for CLOUD are close to those of Wrenformer under the stability threshold of 0, as shown in Table S4.

Table S3: Results on MatBench regression tasks. All results are shown in the mean of test MAE in five-fold cross-validation along with the standard deviation in brackets. The best and the second best results among structure-agnostic and coordinate-free models are in bold and underlined, and the best results among all the models are in italics.

Model	jdft2d (↓)	phonons (↓)	dielectric (↓)	gvrh (↓)	kvrh (↓)	perovskites (↓)	band gap (↓)	e form (↓)
coGN	37.1652 (13.6825)	29.7117 (1.9968)	0.3088 (0.0859)	0.0689 (0.0009)	0.0535 (0.0028)	*0.0269 (0.0008)*	*0.1559 (0.0017)*	*0.0170 (0.0003)*
coNGN	36.1698 (11.5973)	*28.8874 (3.2840)*	0.3142 (0.0740)	*0.0670 (0.0006)*	*0.0491 (0.0026)*	0.0290 (0.0011)	0.1697 (0.0035)	0.0178 (0.0004)
ALIGNN	43.4244 (8.9491)	29.5385 (2.1148)	0.3449 (0.0871)	0.0715 (0.0006)	0.0568 (0.0028)	0.0288 (0.0009)	0.1861 (0.0030)	0.0215 (0.0005)
CGCNN	49.2440 (11.5865)	57.7635 (12.3109)	0.5988 (0.0833)	0.0895 (0.0016)	0.0712 (0.0028)	0.0452 (0.0007)	0.2972 (0.0035)	0.0337 (0.0006)
CrabNet	45.6104 (12.2491)	55.1114 (5.7317)	0.3234 (0.0714)	0.1014 (0.0017)	0.0758 (0.0034)	0.4065 (0.0069)	0.2655 (0.0029)	0.0862 (0.0010)
Finder	47.9614 (11.6681)	46.5751 (3.7415)	0.3204 (0.0811)	0.0996 (0.0018)	0.0764 (0.0025)	0.6450 (0.0167)	0.2308 (0.0029)	0.0839 (0.0011)
Roost	44.6405 (11.7353)	54.3893 (4.7283)	0.3252 (0.0780)	0.1034 (0.0020)	0.0797 (0.0042)	0.4025 (0.0077)	0.2571 (0.0055)	0.0847 (0.0016)
Wrenformer	39.6309	92.3349	0.3583	0.1070	0.0811	0.3351	0.2986	0.0694
MatInFormer	45.1309 (12.7339)	44.0134 (5.7265)	0.3046 (0.0765)	0.0873 (0.0019)	0.0703 (0.0030)	0.4339 (0.0105)	0.2541 (0.0020)	0.0646 (0.0003)
SLICES-BERT	37.8586 (10.9928)	44.5470 (4.0192)	0.3417 (0.0918)	0.0932 (0.0015)	0.0698 (0.0021)	**0.0351 (0.0013)**	0.2776 (0.0043)	0.0543 (0.0004)
CLOUD	*35.0057 (11.4550)*	**40.6856 (2.6168)**	*0.3038 (0.0782)*	**0.0873 (0.0028)**	**0.0682 (0.0026)**	0.0969 (0.0010)	**0.2126 (0.0030)**	**0.0542 (0.0007)**

Table S4: Classification and regression metrics for models tested on MatBench Discovery ranked by F1 score. The stability threshold for model predictions are set to 0.

Model	F1 (↑)	DAF (↑)	Prec (↑)	Acc (↑)	TPR (↑)	TNR (↑)	MAE (↓)	RMSE (↓)	R^2 (↓)
SevenNet	0.719	3.804	0.653	0.893	0.800	0.912	0.046	0.090	0.750
MACE	0.668	3.400	0.583	0.867	0.781	0.885	0.055	0.099	0.698
CHGNet	0.612	3.038	0.521	0.839	0.740	0.859	0.061	0.100	0.690
M3GNet	0.576	2.647	0.454	0.802	0.788	0.804	0.072	0.115	0.588
ALIGNN	0.565	2.921	0.501	0.829	0.649	0.866	0.092	0.154	0.274
MEGNet	0.513	2.699	0.463	0.813	0.574	0.862	0.128	0.204	-0.277
CGCNN	0.510	2.631	0.451	0.807	0.587	0.852	0.135	0.229	-0.624
CGCNN+P	0.510	2.398	0.411	0.779	0.670	0.801	0.108	0.178	0.027
Wrenformer	0.479	2.130	0.365	0.741	0.693	0.751	0.105	0.182	-0.020
CLOUD	0.474	2.043	0.340	0.711	0.781	0.697	0.104	0.167	0.147
BOWSR	0.437	1.836	0.315	0.702	0.711	0.680	0.114	0.164	0.142
Voronoi RF	0.344	1.509	0.259	0.665	0.511	0.697	0.141	0.206	-0.316
Dummy	0.194	1	0.168	0.680	0.231	0.770	0.120	0.181	0

Table S5: Classification metrics for CLOUD tested on MatBench Discovery under varying stability thresholds.

Threshold	F1 (↑)	DAF (↑)	Prec (↑)	Acc (↑)	TPR (↑)	TNR (↑)
-0.05	0.467	2.903	0.484	0.828	0.451	0.904
0.0	0.474	2.043	0.340	0.711	0.781	0.697
0.05	0.380	1.434	0.239	0.494	0.932	0.407

We list the classification results by CLOUD under different stability thresholds in Table S5. Note that the true labels for the test data are derived with the threshold of 0, consistent with the benchmark setting [11], while the dynamic threshold applies to the model prediction. More negative thresholds will result in higher precision for CLOUD and subsequently higher DAF, which is also observed for models that are more optimistic in stability predictions like CHGNet [11].. However, the trade-off across metrics leads to decreased F1 score and TPR when a negative threshold is used.

In order to showcase the potential of CLOUD in material discovery tasks, we further carry out a prospective discovery-style evaluation to directly demonstrate CLOUD's ability to screen for new stable materials. On the WBM dataset which contains material structures that are out-of-distribution (OOD) to the training data, we calculate the energy above hull (E_{hull}), so far the same as the evaluation for MatBench Discovery. We rank all test candidates by their predicted distance to hull, $E_{\text{hull}}^{\text{pred}}$, in ascending order. Fix the stability threshold τ (e.g., 0, 0.02, or 0.05 eV/atom) and define binary labels

$$s_i \;=\; \mathbf{1}\big[E_{\text{hull},i}^{\text{true}} \le \tau\big] \in \{0,1\}, \tag{3}$$

for $i = 1, \ldots, N$. Let π denote the permutation that sorts candidates by $E_{\text{hull}}^{\text{pred}}$ (best first), so $\pi(1)$ is rank-1, etc. We then quantify discovery efficiency under realistic screening budgets using three complementary metrics:

- **Precision@k**: the fraction of true stables in the top-k suggestions,

$$\text{P@}k \;=\; \frac{1}{k}\sum_{j=1}^{k} s_{\pi(j)}. \tag{4}$$

- **Enrichment Factor** at top $\alpha\%$ (with $k = \lfloor \alpha N \rfloor$): the fold improvement over random screening,

$$\text{EF}_{\alpha} \;=\; \frac{1}{k}\sum_{j=1}^{k} s_{\pi(j)} \Big/ \frac{1}{N}\sum_{i=1}^{N} s_i \tag{5}$$

- **Cost-to-M-stable**: the number of evaluations needed to uncover M true stables when following the ranked list,

$$\text{Cost-to-M} \;=\; \min\left\{ m \;:\; \sum_{j=1}^{m} s_{\pi(j)} \;\ge\; M \right\}. \tag{6}$$

For comparison to the theoretical optimum, we also report the cost factor Cost-to-M/$M \geq 1$, where 1 corresponds to the ideal case.

The workflow resembles the true material discovery process where the most promising candidates, rather than the whole pool, are sorted out for final evaluation with theoretical calculation or experiments. As shown in Figure S2**a**, the precision for stability screening remains high even up to the top-1000 candidates and reaches nearly 100% for $k \leq 200$, showing that the model consistently prioritizes true stable materials at the top of the ranked list. The enrichment factor further demonstrates that the model concentrates stable compounds effectively, with as much as ∼10-fold enrichment over random selection within the top fraction of candidates selected by the model (Figure S2**b**). The cost factor curves reveal that CLOUD identifies a target number of stable materials with only about 1.1–1.3 times the evaluations required by the theoretical optimal (Figure S2**c**), highlighting its efficiency under practical screening budgets.

Besides the metrics shown above, we pick one example from the WBM dataset, which is Al_2Fe (space group: No.139, I4/mmm), and compare it with the convex hull for the Al-Fe system obtained from Materials Project [32]. As shown in Figure S2, Al_2Fe, though not included in the Materials Project database, exhibits a lower formation energy than the existing convex hull. Our model CLOUD successfully identifies its stability, despite that the formation energy prediction itself is not perfectly accurate. Therefore, this case study demonstrates how CLOUD can highlight previously unseen stable materials from only symmetry-consistent templates and composition, with unrelaxed structures as input only, thereby reducing the number of expensive high-fidelity evaluations needed for discovery.

S3.3 UnconvBench

Table S6 summarizes the model performance in terms of MAE on UnconvBench to evaluate the predictive performance for 'unconventional' crystals, which are in fact the prevailing crystals in the real world. We provide the average MAE as well as the standard deviation on five folds for each task. CLOUD demonstrates comparable performance to the SOTA model CrysToGraph and significantly outperforms the other structure-based models.

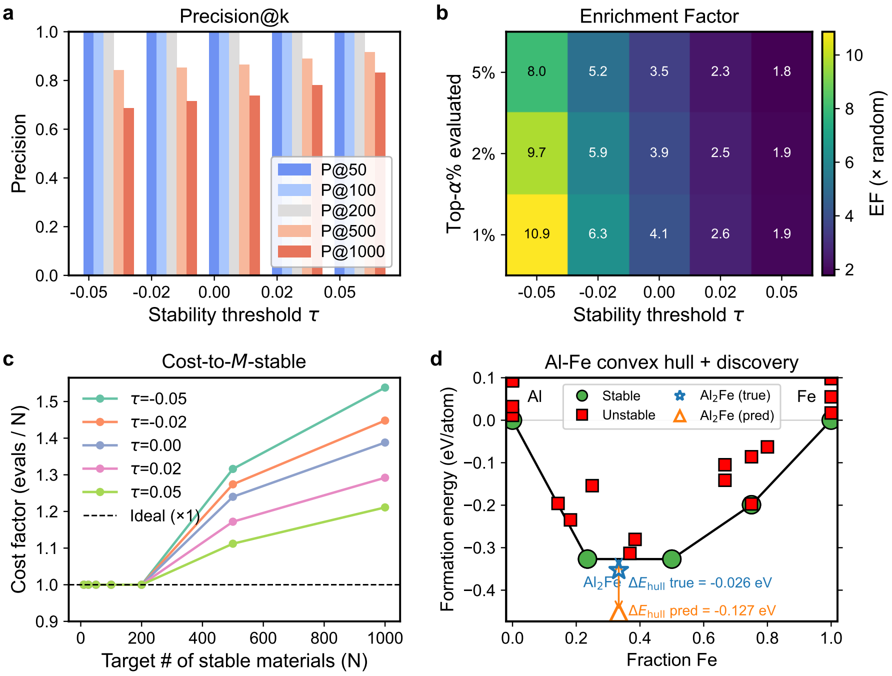

Fig. S2: Evaluation of CLOUD in materials discovery tasks on the WBM dataset. We vary the stability threshold $\tau \in \{-0.05, -0.02, 0.0, 0.02, 0.05\}$ and calculate the following metrics: **a** Precision@k: fraction of true stables recovered within the top-k predictions across stability thresholds; **b** Enrichment Factor: fold enrichment over random screening in the top $\alpha\%$ of the ranked list; **c** Cost-to-M-stable: number of evaluations required to uncover M stable materials, normalized by the ideal case (dashed line). **d** Case study: Al–Fe convex hull. CLOUD correctly identifies the intermetallic Al_2Fe as a new stable candidate.

Table S6: Results on UnconvBench general predictive tasks. All results are shown in the mean of test MAE in five-fold cross-validation. The best results are in bold and the second-best results are underlined. The standard deviations are shown in brackets.

Type	Model	2d_e_exf (↓)	2d_e_tot (↓)	2d_gap (↓)	qmof (↓)	supercon (↓)	defected (↓)
Structure-based	CrysToGraph	**0.0500 (0.0016)**	**0.3623 (0.0210)**	0.0986 (0.0117)	**121.9662 (2.001)**	**2.6422 (0.1722)**	0.8885 (0.1641)
	coGN	0.0510 (0.0020)	0.5214 (0.1278)	0.1168 (0.0178)	218.9272 (10.640)	2.8955 (0.1522)	1.0615 (0.1268)
	coNGN	0.0530 (0.0026)	0.4497 (0.1091)	0.1432 (0.0197)	229.1948 (12.468)	2.9167 (0.0940)	1.0441 (0.1539)
	ALIGNN	0.0580 (0.0031)	0.3705 (0.0743)	0.1048 (0.0145)	217.2508 (6.738)	2.7372 (0.1069)	0.9842 (0.1325)
	CGCNN	0.0710 (0.0053)	1.2941 (0.1208)	0.1499 (0.0209)	231.1887 (8.2983)	2.9316 (0.1069)	1.1321 (0.1100)
Coordinate-free	CLOUD	0.0569 (0.0054)	0.3723 (0.0286)	**0.0919 (0.0070)**	159.5723 (9.6317)	2.6925 (0.1337)	**0.8197 (0.1093)**

Table S7: MAE Comparison between CLOUD and PMCGNN as well as other baselines on JARVIS Dataset. The results for CGCNN, ALIGNN, Matformer, and PMCGNN are directly obtained from Feng et al. [34].

Model	formation energy ev/atom	bandgap (OPT) ev	total energy ev/atom	bandgap (MBJ) ev	E_{hull} eV
CGCNN	0.063	0.20	0.078	0.41	0.17
ALIGNN	0.0331	0.142	0.037	0.31	0.076
Matformer	<u>0.0325</u>	<u>0.137</u>	<u>0.035</u>	<u>0.30</u>	0.064
PMCGNN	**0.0278**	**0.122**	**0.029**	**0.25**	<u>0.040</u>
CLOUD	0.0606	0.159	0.069	0.31	**0.030**

S3.4 JARVIS-DFT

Additionally, we have also fine-tuned CLOUD on JARVIS-DFT-2021 3D dataset [33]. We chose five crystal property prediction tasks: formation energy, bandgap (OPT), total energy, bandgap(MBJ), and E_{hull} for benchmarking. We follow the same data-splitting strategy as described in Feng et al. [34], and compare our model performance with the following baselines: CGCNN [18], ALIGNN [17], Matformer [35], and PMCGNN [34]. As shown in Table S7, our model CLOUD achieves competitive predictive performance, consistently outperforming CGCNN across the five tasks and exhibits the state-of-the-art (SOTA) performance on the E_{hull} dataset. For 4 out of the 5 tasks, PMCGNN stands out with the lowest MAE, reflecting its tailored graph-based inductive bias. Nevertheless, CLOUD reaches comparable accuracy while being coordinate-free and symmetry-consistent by design, highlighting its complementary strengths. In particular, achieving the best performance on E_{hull} demonstrates CLOUD's capability for stability predictions, which are of central importance for materials discovery.

S3.5 Extension of CLOUD-DEBYE: Towards Broader Applications

We have extended our CLOUD-DEBYE framework for predicting vibrational entropy (S_{vib}) to showcase its broad application in many other property prediction tasks. Table S8 summarizes the results for predicting S_{vib}. Similar to our implementations for predicting constant-volume heat capacity C_v and phonon internal energy U, the train and test data are under 300K, and we use MAE/MAD as the metric for comparison. CLOUD-DEBYE outperforms the two descriptor-hybridized GNNs (de-CGCNN and de-ALIGNN) and CLOUD, which is consistent with the observations for C_v and U.

In addition, we extrapolate the model fine-tuned on 300K data to a wide range of temperatures and compare the model predictions to experimental [36–39] as well as phonon calculation results [40]. Similar to the observations for C_v, our model manifests superior predictive accuracy from 0K up to near the melting point of the solid, maintaining physical consistency in model predictions (Figure S3).

Our experimental results present a compelling demonstration of the significance of integrating CLOUD with physical models for physics-consistent predictions. Moreover,

Table S8: Results on predicting S_{vib}. All results are shown in the mean of test MAE/MAD along with the standard deviation in brackets. The best results are in bold and the second-best results are underlined.

Model	S_{vib} (↓)
de-CGCNN	0.154 (0.003)
de-ALIGNN	0.195 (0.005)
CLOUD	0.212 (0.009)
CLOUD-DEBYE	**0.121 (0.004)**

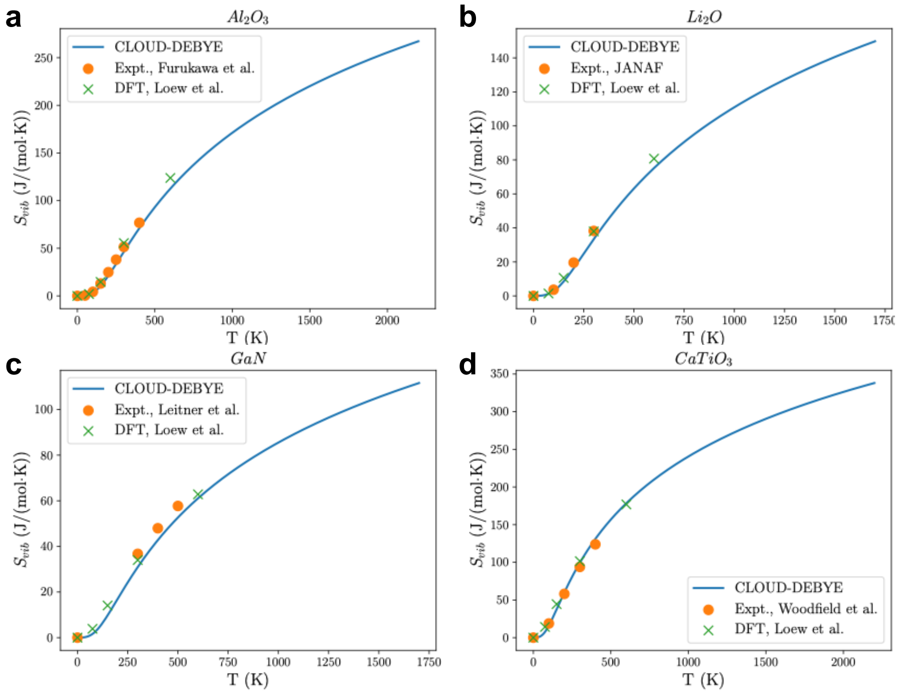

Fig. S3: S_{vib} predictions by CLOUD-DEBYE for **a** Al_2O_3, **b** Li_2O, **c** $CaTiO_3$, and **d** GaN. The CLOUD-DEBYE model, fine-tuned on S_{vib} data at 300K, accurately extrapolates heat capacity across a wide temperature range, approaching the melting point of each material. Model predictions are compared against experimental and DFT-calculated results.

our framework of embedding physics-based, differentiable laws into machine learning models is not limited to the Debye model. In principle, this approach can be generalized to any physical system, provided that the underlying physics can be implemented in a

differentiable manner and sufficient data are available to learn the complex mappings that remain intractable in the physics model. In the following, we will briefly discuss the possible extensions of the CLOUD-physics framework which will serve as our future work:

- **Phonon-related properties from pDOS** While the Debye model provides a quadratic approximation to the phonon spectrum and thus misses optical branches and fine spectral features, these can be captured by predicting the full phonon density of states (pDOS). In this setting, the ML model predicts the pDOS $g(\omega)$ directly from the crystal representation, which is then fed into analytic statistical–mechanics relations for vibrational thermodynamics. For example, the constant–volume heat capacity C_v is given by

$$C_v(T) = \int_0^\infty \hbar\omega\, g(\omega) \left[\frac{1}{\left(e^{\hbar\omega/k_BT} - 1\right)^2} e^{\hbar\omega/k_BT} \frac{\hbar\omega}{k_BT^2} \right] d\omega, \tag{7}$$

  and the vibrational entropy by

$$S_{\text{vib}}(T) = k_B \int_0^\infty g(\omega) \left[\frac{\hbar\omega/k_BT}{e^{\hbar\omega/k_BT} - 1} - \ln\left(1 - e^{-\hbar\omega/k_BT}\right) \right] d\omega. \tag{8}$$

  Therefore, the mapping from pDOS to thermodynamic observables is fully differentiable, allowing gradients to backpropagate through the integrals, and (ii) a single ML–predicted $g(\omega)$ provides access to multiple vibrational quantities (e.g. C_v, S_{vib}, free energy), enabling multitask training and richer physics constraints.
- **Elastic modulus**: Elastic properties can be described by closed-form averages of the elastic tensor C_{ij}. The Voigt bounds depend linearly on C:

$$K_V = \frac{1}{9}\left(C_{11} + C_{22} + C_{33} + 2(C_{12} + C_{23} + C_{13})\right) \tag{9}$$

$$G_V = \frac{1}{15}\left[(C_{11} + C_{22} + C_{33} - C_{12} - C_{23} - C_{13}) + 3(C_{44} + C_{55} + C_{66})\right] \tag{10}$$

  The Reuss bounds depend on the compliance tensor $S = C^{-1}$:

$$K_R = \frac{9}{S_{11} + S_{22} + S_{33} + 2(S_{12} + S_{23} + S_{13})} \tag{11}$$

$$G_R = \frac{15}{4(S_{11} + S_{22} + S_{33}) - 4(S_{12} + S_{23} + S_{13}) + 3(S_{44} + S_{55} + S_{66})} \tag{12}$$

  Therefore, the Voigt–Reuss–Hill averages are

$$K_{VRH} = \tfrac{1}{2}(K_V + K_R), \qquad G_{VRH} = \tfrac{1}{2}(G_V + G_R) \tag{13}$$

  All mappings are smooth wherever C is positive-definite, ensuring gradients propagate K_{VRH} or G_{VRH} back to update the model parameters.

- **Thermal expansion coefficient**: The thermal expansion coefficient relates to thermodynamic quantities through the Grüneisen parameter γ and temperature-dependent bulk modulus [41]:

$$\alpha(T) = \frac{\gamma C_v(T)}{K_T(T) V_{\text{molar}}} \tag{14}$$

where $C_v(T)$ is the heat capacity from the Debye model and the bulk modulus varies with temperature according to:

$$K_T(T) = K_0(1 - \alpha_K T) \tag{15}$$

The linear thermal expansion follows:

$$\frac{\Delta L}{L_0} = \int_{T_0}^{T} \alpha(T') dT' \tag{16}$$

The model predicts the zero-temperature bulk modulus K_0, its temperature coefficient α_K, and the Grüneisen parameter γ. Since all operations are smooth and the thermal expansion coefficient depends differentiably on these predicted quantities, gradients flow seamlessly from experimental thermal expansion measurements back to optimize the model parameters for representation learning.

Together, these examples illustrate the breadth of our framework: CLOUD–pDOS captures full vibrational spectra for C_v and S_{vib}, VRH averaging provides smooth and bounded mappings for elastic modulus, and thermodynamic relations yield temperature-dependent thermal expansion. These cases highlight the broader promise of combining CLOUD with physical laws wherever sufficient training data are available.

S3.6 Robustness Analysis

In addition to the property prediction results, we investigate the robustness of building SCOPE representations and CLOUD model predictions under controlled perturbations of the input crystal structures. Such perturbations are practically unavoidable in realistic materials research, arising from geometry optimization tolerances, noise in machine-learned force fields, or the thresholds used by symmetry analyzers. Because the SCOPE representation relies on symmetry parsing, even small distortions can alter the assigned space group or Wyckoff positions. We therefore probe how predictive accuracy and parsing stability degrade under systematically applied noise to atomic coordinates and lattice vectors, providing a quantitative measure of the sensitivity of our models to structural perturbations.

A recent independent study by Siron et al. [42] provides an external benchmark of this issue. They systematically compared multiple structure identification and fingerprinting approaches—including the SCOPE represention for CLOUD, Pymatgen's StructureMatcher, SLICES, and their proposed BAWL hashing—for matching perturbed crystal structures. Perturbations included Gaussian noise on fractional atomic

coordinates and on lattice vectors, as well as symmetry operations and translations. Their results show that SCOPE (and thus CLOUD) maintains moderate robustness to small perturbations, outperforming other representations such as SLICES, while specialized perturbation-invariant methods such as BAWL achieve higher tolerance at larger noise levels. This analysis establishes that SCOPE is reasonably stable under minor structural distortions.

Building on this prior evaluation, we additionally perform a dedicated robustness study of our model predictions by perturbing each test crystal with (i) fractional–coordinate noise, adding i.i.d. $\mathcal{N}(0,\sigma^2)$ to fractional positions, and (ii) lattice strain, drawing a random symmetric small-strain tensor ε with i.i.d. entries $\mathcal{N}(0,\sigma^2)$, symmetrizing $\varepsilon = \frac{1}{2}(E + E^\top)$; this perturbs both lattice lengths and angles (normal and shear). For every perturbed structure we regenerate SCOPE and evaluate fixed checkpoints (no retraining) over a log grid σ from 10^{-4} up to at most 10^0. Alongside prediction error (Figure S4**a-c**) we report the symmetry-parse success rate (Figure S4**d**). On the gvrh dataset from MatBench [12], the MAE for unperturbed structures is 0.0873 ± 0.0028. Fractional coordinate noise induces a monotonic increase—0.1004 at $\sigma = 3 \times 10^{-4}$ (+15%) and 0.1201 at 10^{-3} (+37.6%)—whereas lattice strain shows much less significant impact on model performance up to 10^{-2} (MAE ≈ 0.090, $\leq +4\%$). Parsing succeeds for all small-σ cases (rate = 1.0 through 10^{-2}); degradation appears only under heavy stress (e.g., at $\sigma = 0.1$ the success rate drops to 0.774 for coordinate noise and 0.987 for strain, and to 0.075 / 0.399 at $\sigma = 0.3$). On the C_v dataset, CLOUD is markedly more sensitive to coordinate noise: $8.92 \rightarrow 11.00$ at 10^{-4} (+23%), 19.71 at 3×10^{-4} (+121%), and 27.04 at 10^{-3} (+203%); strain grows more gently (12.29 at 10^{-3}, 13.66 at 10^{-2}). Symmetry parse success remains ≈ 0.997 up to 10^{-2}, indicating that our method of constructing SCOPE for crystal structures is robust to small perturbation levels. Adding the physics head in CLOUD–DEBYE both lowers baseline error (3.19 ± 0.04) and dampens sensitivity: under coordinate noise at $\sigma = 10^{-3}$ the MAE is 5.27 (versus 27.04 for CLOUD), and under strain at $\sigma = 10^{-2}$ it is 4.66 (versus 13.66). Meanwhile, we need to point out that the symmetry parsing success does not necessarily guarantee that the true symmetry for the unperturbed structure could be recovered. Overall, these results demonstrate that CLOUD is reasonably robust to modest structural perturbations, with lattice strain having limited effect and coordinate noise being more impactful, while the physics-informed CLOUD–DEBYE variant substantially mitigates sensitivity and enhances stability across perturbation regimes.

S3.7 Ablation Studies

To systematically evaluate the design choices underlying our SCOPE representation, we conduct a series of ablation studies across multiple datasets. Specifically, we vary (i) the notation used to encode space group symmetry—numerical index (1–230), Hermann–Mauguin (HM) symbols, or generator strings (GS) of symmetry operations—and (ii) whether Wyckoff positions (WPs) are included. We also examined a composition-only baseline, where symmetry information is ignored entirely, to assess the added value of structural encodings. For each variant, we pre-train CLOUD and fine-tune the resulting models on four representative benchmark datasets: `dielectric`

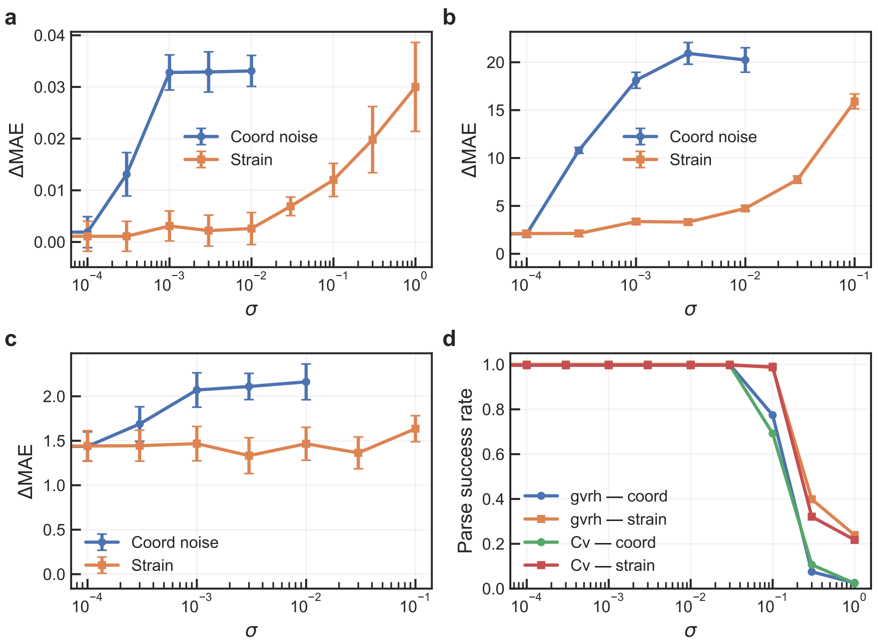

Fig. S4: Model performance robustness analysis. Gaussian noise with varying σ's are added to the atomic positions or lattice strain applied to lattice vectors. We show the ΔMAE due the noise for **a** gvrh with CLOUD model; **b** Cv with CLOUD model; **c** Cv with CLOUD-DEBYE model. In addition, the symmetry parsing successful rate under different noises are presented in **d**.

and `e form` from MatBench [12], the `defected` dataset from UnconvBench [14], and the C_v dataset from Gong et al. [15]. These tasks span both conventional property prediction benchmarks and settings where long-range interactions or structural disorder are especially relevant.

The results, summarized in Table S9, yield several consistent trends. First, across the MatBench tasks, including WPs consistently improve accuracy regardless of the space group notation, with generator strings plus WPs achieving the best performance overall. This indicates that both generator-level tokens and fine-grained Wyckoff position information are crucial in modeling the material structures. Meanwhile, for the `defected` dataset, generator strings again provide the strongest symmetry encoding, but the inclusion of WPs slightly reduces performance. This can be attributed to the dataset construction: vacancies are introduced at unique Wyckoff sites without explicit annotation of defect location [43], making WP tokens a source of noise rather than signal. Finally, in the C_v task, the composition-only baseline performs poorly, underscoring the insufficiency of stoichiometry alone for material representation learning. Incorporating symmetry information markedly improves accuracy, with generator strings delivering the lowest errors. Together, these findings quantitatively validate

Table S9: Ablation study on the SCOPE representation across four datasets with different variants of SCOPE design. Results are reported as the mean test MAE for `dielectric`, `e form`, and `defected` and MAE/MAD for C_v (and standard deviation) from five-fold cross-validation. Bold indicates the best performance within each dataset, and underline denotes the second-best.

SG notation	WP	dielectric (↓)	e form (↓)	defected (↓)	C_v (↓)
N/A	✗	0.3438 (0.0916)	0.0845 (0.0007)	0.8544 (0.1894)	0.48 (0.01)
Index	✗	0.3551 (0.0974)	0.0599 (0.0006)	<u>0.7769 (0.1946)</u>	0.17 (0.00)
	✓	0.3345 (0.0806)	<u>0.0568 (0.0004)</u>	0.9360 (0.1897)	0.17 (0.01)
HM	✗	0.3333 (0.0751)	0.0604 (0.0003)	0.8188 (0.1890)	0.18 (0.00)
	✓	0.3290 (0.0894)	0.0569 (0.0008)	0.8257 (0.1539)	**0.16 (0.01)**
GS	✗	<u>0.3289 (0.0771)</u>	0.0601 (0.0006)	**0.7482 (0.1648)**	0.17 (0.01)
	✓	**0.3038 (0.0782)**	**0.0542 (0.0007)**	0.8197 (0.1093)	**0.16 (0.00)**

the benefit of generator-level symmetry encoding while also clarifying the contexts in which Wyckoff positions provide complementary information.

In summary, these ablation experiments confirm that symmetry information encoded through generator strings is consistently advantageous across datasets. The inclusion of Wyckoff positions further enhances performance when their meaning is well defined, whereas it may degrade performance in defected structures where such information becomes ambiguous. Overall, the combination of generator-level encodings and Wyckoff information provides the most effective and generalizable representation of crystal symmetry within SCOPE.

S3.8 Training Efficiency and Scalability

The computational cost of large-scale pre-training is an important consideration for practical application of foundation models. For the results reported in this work, we pre-train CLOUD on the deduplicated OPTIMADE dataset (∼6.3M sequences). Using two NVIDIA H100 GPUs, the base model (∼110M parameters) requires ∼10 minutes per epoch. Such a cost is modest compared to other large-scale machine learning models in materials science and is further mitigated by the fact that pre-training is a one-time investment. Once obtained, a pretrained checkpoint can be reused across diverse fine-tuning tasks, consequently amortizing the initial cost over a broad range of downstream applications.

To evaluate the scaling efficiency of CLOUD on the Aurora HPC, the exascale supercomputer at Argonne National Laboratory, we measure the model throughput while scaling the number of nodes for two versions of the CLOUD model: a 110M parameter baseline and a 1B parameter larger model. The weak scaling setup fixes the local batch size per GPU, allowing us to isolate the effect of communication overhead as the cluster size grows. As shown in Figure S5a, both models sustain high efficiency up to 128 nodes, maintaining efficiency higher than 60%. Notably, the 1B model consistently outperforms the 110M model in scalability, particularly at higher node counts,

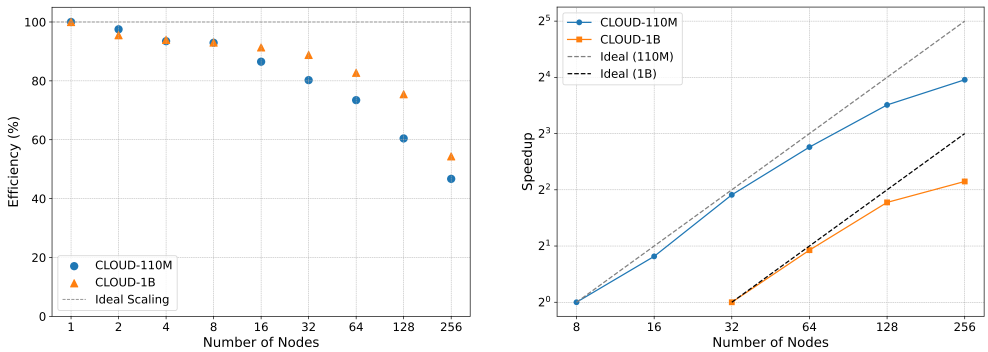

(a) Scaling Efficiency vs. Number of Nodes for training CLOUD with 110M and 1B parameter models on Aurora using our training stack. Efficiency is computed under weak scaling by fixing the local batch size per GPU. Both models maintain high parallel efficiency up to 128 nodes (higher than 60%), with the 1B model exhibiting better scalability at large node counts due to improved compute-to-communication ratio.

(b) Scaling Speedup vs. Number of Nodes for training CLOUD with 110M and 1B parameter models on Aurora using our training stack. Speedup is computed relative to the smallest measured node count (8 for 110M, 32 for 1B), with ideal scaling shown as dashed lines. Both the two models manifest near-perfect strong scaling behavior up to 64 nodes for 110M model or 128 nodes for 1B model.

Fig. S5: Scalability analysis for CLOUD on Aurora [44].

benefiting from a larger compute footprint that better amortizes communication overhead. We evaluate the strong scaling behavior of the CLOUD model on Aurora for the two versions of model. In this setup, the total training workload remains constant as the number of nodes increases, and we record the reduction in runtime to compute speedup. As shown in Figure S5b, the 110M model shows near-linear speedup up to 64 nodes and reasonable scaling beyond. The 1B model, evaluated from 32 to 128 nodes, demonstrates consistent gains, though with diminishing returns at higher scales due to communication overhead. The log-log plots highlight the efficiency of distributed training, validating our stack's readiness for exascale foundation model training.

Furthermore, compared to graph-based models that require explicit 3D atomic coordinates or costly DFT relaxations, CLOUD substantially lowers the end-to-end computational burden for large-scale discovery workflows. Most state-of-the-art models for material structure modeling are based on graph neural networks (GNNs), which face intrinsic scaling challenges such as oversmoothing and oversquashing [45]. In addition, distributed training of GNNs requires graph partitioning, where boundary nodes and edges induce significant cross-device communication overhead. In contrast, transformers benefit from well-established parallelization strategies that scale efficiently with modern hardware.

In addition to pre-training efficiency, the practicality of CLOUD also depends on the cost of fine-tuning and inference. To further assess computational efficiency in practical settings, we compared training and inference times of CLOUD against widely

Table S10: Training and inference time comparison on material property prediction tasks. Reported numbers include training time per epoch, inference time on the full dataset (for Jarvis formation energy task) or the test set (for MatBench formation energy task), and model size.

Model	Time/epoch	Inference	Parameters
ALIGNN (Jarvis FE)	327 s	156 s	15.4 M
Matformer (Jarvis FE)	64 s	59 s	11.0 M
CLOUD (Jarvis FE)	34 s	19 s	110 M
MEGNet (MatBench FE)	N/A	11.137 s	168 K
M3GNet (MatBench FE)	N/A	20.089 s	228 K
TensorNet (MatBench FE)	N/A	13.694 s	2.8 M
SO3Net (MatBench FE)	N/A	32.601 s	341 K
CLOUD (MatBench FE)	59.892 s	7.003 s	110 M

used GNN baselines (ALIGNN [17], Matformer [35], MEGNet [27], M3GNet [26], TensorNet [46], and SO3Net [47]) on representative property prediction tasks (Jarvis [33] and MatBench [12] formation energy datasets). The results for ALIGNN and Matformer on Jarvis formation energy task are obtained from Yan et al. [35] while the numbers for the MatBench formation energy task are collected from implementations with MatGL [48]. As summarized in Table S10, CLOUD achieves faster training time per epoch and significantly shorter inference time despite being larger in model parameter count. For example, on the Jarvis FE task, CLOUD requires only 34 seconds per epoch and 19 seconds for full inference, outperforming ALIGNN and Matformer while offering an order-of-magnitude faster inference throughput. Similarly, on the MatBench FE benchmark, CLOUD completes inference in only 7 seconds on the test set, the fastest among all tested models. These results highlight that the transformer-based architecture not only scales efficiently during pre-training but also provides lightweight fine-tuning and inference in downstream applications.

For baseline comparisons, we follow the MatBench protocol where CGCNN and ALIGNN are trained from scratch in a supervised manner, without pre-training. While ALIGNN provides pre-trained checkpoints, these are obtained via supervised pre-training on labeled data such as energies and forces, in contrast to the self-supervised strategy adopted for CLOUD. Attempts to develop self-supervised pre-training for GNNs, such as the Crystal Twins framework [49], have demonstrated only modest and inconsistent improvements over supervised CGCNN and remain less effective than ALIGNN. We further apply the Crystal Twins framework to pre-train CGCNN on the same $\sim$6.3M crystal structures as CLOUD, but find that the pre-training process is prohibitively slow: after eight hours, only $\sim$12% of the training steps in one epoch are completed, compared to $\sim$10 minutes per epoch for CLOUD. We acknowledge that our implementation may not be fully optimized, but the evidence nevertheless indicates that self-supervised pre-training for GNNs is still far from mature. The comparative results on the MatBench GVRH dataset (Table S11) further support this point: while

CGCNN with Crystal Twins pre-training achieves only marginal gains over its supervised counterpart, CLOUD demonstrates a substantial and consistent improvement when pre-trained. These observations highlight both the limited effectiveness and poor scalability of GNN-based pre-training, underscoring why transformer-based models like CLOUD are particularly well-suited for large-scale self-supervised learning with crystals.

Table S11: Comparison of model performance on the MatBench gvrh dataset. Results are reported as the mean of five-fold MAE with standard deviations. Results for CGCNN (supervised) and Crystal Twins variants are taken from [49]; CLOUD and replicated CGCNN-CT results are from this work.

Model	Pre-training	MAE ± std
CGCNN [49]	None (supervised)	0.089 ± 0.001
CGCNN (Crystal Twins) [49]	SSL (CTBarlow)	0.086 ± 0.004
CGCNN (this work)	SSL (6.3M, CT code)	0.092 ± 0.004
CLOUD (this work)	None (supervised)	0.117 ± 0.003
CLOUD (this work)	SSL (6.3M, MLM)	0.087 ± 0.003

S3.9 Attention Visualization

We visualize the attention score between the [CLS] token and the other tokens in SCOPE from different hidden layers and attention heads in Figure S6, using NaI from the C_v dataset as an example. While [CLS] tends to attend to different tokens in different attention heads, which is expected as different attention heads are meant to capture features in different subspaces, some tokens are intensively attended across attention heads which are highlighted in red at the bottom of the figure. Aside from material compositions, space group tokens also have relatively high attention scores with [CLS], which partly explains why CLOUD exceeds GNNs in learning features of global crystal structures and achieving high accuracy in predicting heat capacity and phonon internal energy.

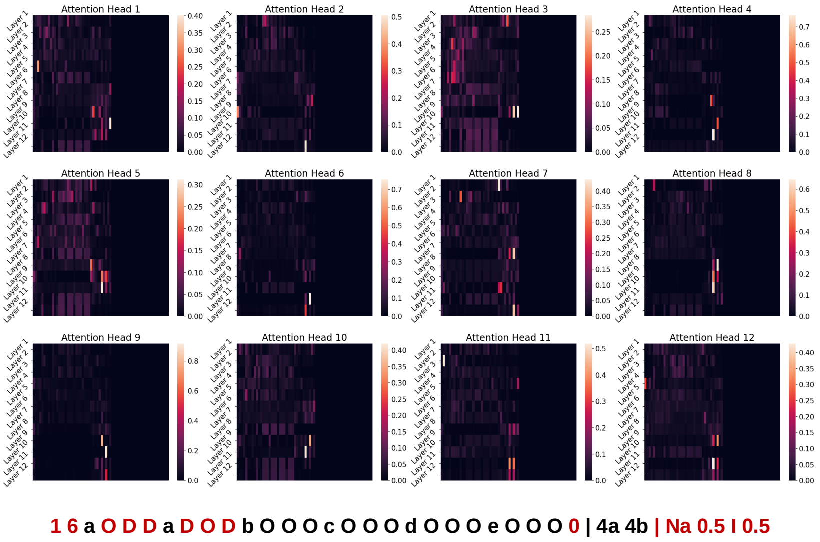

Fig. S6: Visualization of the attention scores between the [CLS] token and the other tokens in different hidden layers and attention heads for NaI example from the C_v test set. The corresponding string representation is put under the plot with the tokens of high attention scores marked in red.

References

[1] Grygorenko, O.O., Radchenko, D.S., Dziuba, I., Chuprina, A., Gubina, K.E., Moroz, Y.S.: Generating multibillion chemical space of readily accessible screening compounds. iScience **23**(11), 101681 (2020) https://doi.org/10.1016/j.isci.2020.101681

[2] Bellmann, L., Penner, P., Gastreich, M., Rarey, M.: Comparison of combinatorial fragment spaces and its application to ultralarge make-on-demand compound catalogs. Journal of Chemical Information and Modeling **62**(3), 553–566 (2022) https://doi.org/10.1021/acs.jcim.1c01378 https://doi.org/10.1021/acs.jcim.1c01378. PMID: 35050621

[3] Patel, H., Ihlenfeldt, W.-D., Judson, P.N., Moroz, Y.S., Pevzner, Y., Peach, M.L., Delannée, V., Tarasova, N.I., Nicklaus, M.C.: Savi, in silico generation of billions of easily synthesizable compounds through expert-system type rules. Scientific Data (2020)

[4] Gavezzotti, A.: Are crystal structures predictable? Accounts of chemical research **27**(10), 309–314 (1994)

[5] Merchant, A., Batzner, S., Schoenholz, S.S., Aykol, M., Cheon, G., Cubuk, E.D.:

Scaling deep learning for materials discovery. Nature, 1–6 (2023)

[6] Zeni, C., Pinsler, R., Zügner, D., Fowler, A., Horton, M., Fu, X., Shysheya, S., Crabbé, J., Sun, L., Smith, J., et al.: Mattergen: a generative model for inorganic materials design. arXiv preprint arXiv:2312.03687 (2023)

[7] Antunes, L.M., Butler, K.T., Grau-Crespo, R.: Crystal structure generation with autoregressive large language modeling. arXiv preprint arXiv:2307.04340 (2023)

[8] Gruver, N., Sriram, A., Madotto, A., Wilson, A.G., Zitnick, C.L., Ulissi, Z.: Fine-tuned language models generate stable inorganic materials as text. arXiv preprint arXiv:2402.04379 (2024)

[9] Barroso-Luque, L., Shuaibi, M., Fu, X., Wood, B.M., Dzamba, M., Gao, M., Rizvi, A., Zitnick, C.L., Ulissi, Z.W.: Open materials 2024 (omat24) inorganic materials dataset and models. arXiv preprint arXiv:2410.12771 (2024)

[10] Deng, B., Zhong, P., Jun, K., Riebesell, J., Han, K., Bartel, C.J., Ceder, G.: Chgnet as a pretrained universal neural network potential for charge-informed atomistic modelling. Nature Machine Intelligence **5**(9), 1031–1041 (2023)

[11] Riebesell, J., Goodall, R.E., Jain, A., Benner, P., Persson, K.A., Lee, A.A.: Matbench discovery–an evaluation framework for machine learning crystal stability prediction. arXiv preprint arXiv:2308.14920 (2023)

[12] Dunn, A., Wang, Q., Ganose, A., Dopp, D., Jain, A.: Benchmarking materials property prediction methods: the matbench test set and automatminer reference algorithm. npj Computational Materials **6**(1), 138 (2020)

[13] Wang, H.-C., Botti, S., Marques, M.A.: Predicting stable crystalline compounds using chemical similarity. npj Computational Materials **7**(1), 12 (2021)

[14] Wang, H., Sun, J., Liang, J., Zhai, L., Tang, Z., Li, Z., Zhai, W., Wang, X., Gao, W., Gong, S.: Crystograph: A comprehensive predictive model for crystal material properties and the benchmark. Battery Energy, 70004 (2024)

[15] Gong, S., Yan, K., Xie, T., Shao-Horn, Y., Gomez-Bombarelli, R., Ji, S., Grossman, J.C.: Examining graph neural networks for crystal structures: limitations and opportunities for capturing periodicity. Science Advances **9**(45), 3245 (2023)

[16] Ruff, R., Reiser, P., Stühmer, J., Friederich, P.: Connectivity optimized nested graph networks for crystal structures. arXiv preprint arXiv:2302.14102 (2023)

[17] Choudhary, K., DeCost, B.: Atomistic line graph neural network for improved materials property predictions. npj Computational Materials **7**(1), 185 (2021)

[18] Xie, T., Grossman, J.C.: Crystal graph convolutional neural networks for an accurate and interpretable prediction of material properties. Physical review letters

120(14), 145301 (2018)

[19] Wang, A.Y.-T., Kauwe, S.K., Murdock, R.J., Sparks, T.D.: Compositionally restricted attention-based network for materials property predictions. Npj Computational Materials **7**(1), 77 (2021)

[20] Ihalage, A., Hao, Y.: Formula graph self-attention network for representation-domain independent materials discovery. Advanced Science **9**(18), 2200164 (2022)

[21] Goodall, R.E., Lee, A.A.: Predicting materials properties without crystal structure: Deep representation learning from stoichiometry. Nature communications **11**(1), 6280 (2020)

[22] Huang, H., Magar, R., Xu, C., Farimani, A.B.: Materials informatics transformer: A language model for interpretable materials properties prediction. arXiv preprint arXiv:2308.16259 (2023)

[23] Xiao, H., Li, R., Shi, X., Chen, Y., Zhu, L., Chen, X., Wang, L.: An invertible, invariant crystal representation for inverse design of solid-state materials using generative deep learning. Nature Communications **14**(1), 7027 (2023)

[24] Park, Y., Kim, J., Hwang, S., Han, S.: Scalable parallel algorithm for graph neural network interatomic potentials in molecular dynamics simulations. Journal of Chemical Theory and Computation (2024)

[25] Batatia, I., Kovacs, D.P., Simm, G., Ortner, C., Csányi, G.: Mace: Higher order equivariant message passing neural networks for fast and accurate force fields. Advances in Neural Information Processing Systems **35**, 11423–11436 (2022)

[26] Chen, C., Ong, S.P.: A universal graph deep learning interatomic potential for the periodic table. Nature Computational Science **2**(11), 718–728 (2022)

[27] Chen, C., Ye, W., Zuo, Y., Zheng, C., Ong, S.P.: Graph networks as a universal machine learning framework for molecules and crystals. Chemistry of Materials **31**(9), 3564–3572 (2019)

[28] Gibson, J., Hire, A., Hennig, R.G.: Data-augmentation for graph neural network learning of the relaxed energies of unrelaxed structures. npj Computational Materials **8**(1), 211 (2022)

[29] Zuo, Y., Qin, M., Chen, C., Ye, W., Li, X., Luo, J., Ong, S.P.: Accelerating materials discovery with bayesian optimization and graph deep learning. Materials Today **51**, 126–135 (2021)

[30] Ward, L., Liu, R., Krishna, A., Hegde, V.I., Agrawal, A., Choudhary, A., Wolverton, C.: Including crystal structure attributes in machine learning models of formation energies via voronoi tessellations. Physical Review B **96**(2), 024104

(2017)

[31] Ward, L., Agrawal, A., Choudhary, A., Wolverton, C.: A general-purpose machine learning framework for predicting properties of inorganic materials. npj Computational Materials **2**(1), 1–7 (2016)

[32] Jain, A., Ong, S.P., Hautier, G., Chen, W., Richards, W.D., Dacek, S., Cholia, S., Gunter, D., Skinner, D., Ceder, G., et al.: Commentary: The materials project: A materials genome approach to accelerating materials innovation. APL materials **1**(1) (2013)

[33] Choudhary, K., Garrity, K.F., Reid, A.C., DeCost, B., Biacchi, A.J., Hight Walker, A.R., Trautt, Z., Hattrick-Simpers, J., Kusne, A.G., Centrone, A., *et al.*: The joint automated repository for various integrated simulations (jarvis) for data-driven materials design. npj computational materials **6**(1), 173 (2020)

[34] Feng, H., Tian, H.: Improving crystal property prediction from a multiplex graph perspective. Journal of Chemical Information and Modeling **64**(19), 7376–7385 (2024)

[35] Yan, K., Liu, Y., Lin, Y., Ji, S.: Periodic graph transformers for crystal material property prediction. Advances in Neural Information Processing Systems **35**, 15066–15080 (2022)

[36] Furukawa, G.T., Douglas, T.B., McCoskey, R.E., Ginnings, D.C.: Thermal properties of aluminum oxide from 0 to 1200 k. Journal of research of the National Bureau of Standards **57**(2), 67–82 (1956)

[37] Stull, D.R.: JANAF Thermochemical Tables... vol. 1. Clearinghouse, Washington, D.C. (1965)

[38] Woodfield, B.F., Shapiro, J.L., Stevens, R., Boerio-Goates, J., Putnam, R.L., Helean, K.B., Navrotsky, A.: Molar heat capacity and thermodynamic functions for catio3. The Journal of Chemical Thermodynamics **31**(12), 1573–1583 (1999)

[39] Leitner, J., Strejc, A., Sedmidubsky, D., Ruzicka, K.: High temperature enthalpy and heat capacity of gan. Thermochimica acta **401**(2), 169–173 (2003)

[40] Loew, A., Sun, D., Wang, H.-C., Botti, S., Marques, M.A.: Universal machine learning interatomic potentials are ready for phonons. arXiv preprint arXiv:2412.16551 (2024)

[41] Grüneisen, E.: Theorie des festen zustandes einatomiger elemente. Annalen der Physik **344**(12), 257–306 (1912)

[42] Siron, M., Djafar, I., Fayet, E., Rossello, A., Ramlaoui, A., Duval, A.: Lemat-bulk: aggregating, and de-duplicating quantum chemistry materials databases. In: AI

for Accelerated Materials Design-ICLR 2025 (2025)

[43] Choudhary, K., Sumpter, B.G.: Can a deep-learning model make fast predictions of vacancy formation in diverse materials? AIP Advances **13**(9) (2023)

[44] Argonne Leadership Computing Facility: Aurora. https://www.alcf.anl.gov/aurora

[45] Wang, Y., Cho, K.: Non-convolutional graph neural networks. arXiv preprint arXiv:2408.00165 (2024)

[46] Simeon, G., De Fabritiis, G.: Tensornet: Cartesian tensor representations for efficient learning of molecular potentials. Advances in Neural Information Processing Systems **36**, 37334–37353 (2023)

[47] Schütt, K.T., Hessmann, S.S., Gebauer, N.W., Lederer, J., Gastegger, M.: Schnetpack 2.0: A neural network toolbox for atomistic machine learning. The Journal of Chemical Physics **158**(14) (2023)

[48] Ko, T.W., Deng, B., Nassar, M., Barroso-Luque, L., Liu, R., Qi, J., Thakur, A.C., Mishra, A.R., Liu, E., Ceder, G., *et al.*: Materials graph library (matgl), an open-source graph deep learning library for materials science and chemistry. npj Computational Materials **11**(1), 253 (2025)

[49] Magar, R., Wang, Y., Barati Farimani, A.: Crystal twins: self-supervised learning for crystalline material property prediction. npj Computational Materials **8**(1), 231 (2022)
